# Methylenetetrahydrofolate reductase gene polymorphism, global DNA methylation and blood pressure: a population based study from North India

**DOI:** 10.1186/s12920-021-00895-1

**Published:** 2021-02-27

**Authors:** Suniti Yadav, Imnameren Longkumer, Shipra Joshi, Kallur Nava Saraswathy

**Affiliations:** 1 Indian Council of Medical Research, New Delhi, 110029 India; 2 Department of Anthropology, University of Delhi, Delhi, 110007 India; 3 Manbhum Ananda Ashram Nityananda Trust-MANT, Kolkata, West Bengal 700078 India

**Keywords:** Hypertension, MTHFR C677T gene polymorphism, Global DNA methylation, Mendelian population, Epigenetics

## Abstract

**Background:**

Hypertension is a complex disorder affected by gene-environment interactions. Methylenetetrahydrofolate reductase (*MTHFR*) gene is one of the genes in One Carbon Metabolic (OCM) pathway that affects both blood pressure and epigenetic phenomenon. *MTHFR* C677T gene polymorphism leads to reduced methylation capacity via increased homocysteine concentrations. Global DNA methylation (5mC%) also gets affected in conditions such as hypertension. However, no study is found to understand hypertension in terms of both genetics and epigenetics. The present study aims to understand the relation between methylation, *MTHFR* C677T gene polymorphism and hypertension. It also tries to understand relation (if any) between methylation and anti-hypertensive drugs.

**Methods:**

This is a cross-sectional study where data were collected from a total of 1634 individuals of either sex in age group 35–65 years. Hypertensives (SBP ≥ 140 mm Hg and DBP ≥ 90 mm Hg) (on treatment/not on treatment) and absolute controls were 236 (cases) and 307 (controls), respectively. All the samples were subjected to *MTHFR* C677T gene polymorphism screening (PCR–RFLP) and global DNA methylation assay (ELISA based colorimetric assay). Results of both the analyses were obtained on 218 cases, 263 controls.

**Results:**

Median 5mC% was relatively lower among cases (p > 0.05) compared to controls, despite controlling for confounders (age, sex, smoking, alcohol, diet) (r^2^-0.92, p-0.08). Cases not on medication had significantly reduced 5mC% compared to controls (p < 0.05), despite adjusting for confounders (r^2^-0.857, p-0.01). Among cases (irrespective of treatment), there was a significant variation in 5mC% across the three genotypes i.e. CC, CT and TT, with no such variation among controls. Cases (not on medication) with TT genotype had significantly lower methylation levels compared to the TT genotype controls and cases (on medication) (p < 0.01).

**Conclusion:**

Global DNA hypomethylation seems to be associated with hypertension and antihypertensive drugs seem to improve methylation. Hypertensive individuals with TT genotype but not on medication are more likely to be prone to global DNA hypomethylation. Important precursors in OCM pathway include micronutrients such as vitamin B-12, B-9 and B-6; their nutritional interventions (either dietary or supplement) may serve as strategies to prevent hypertension at population level. However, more epidemiological-longitudinal studies are needed for further validation.

## Background

Hypertension or elevated blood pressure is a complex disorder affected by an interaction between genes and environment [1]. Till late 1990′s, hypertension was considered to be majorly influenced by genes [2]. The genes so involved in several pathways affect hypertension either directly or indirectly through other downstream metabolites. Methylenetetrahydrofolate reductase (*MTHFR*) gene is one such gene that affects human hypertension. Several studies have been conducted to understand the association of *MTHFR* C677T gene polymorphism with hypertension, but with variable results [3–6]. *MTHFR* gene is also the gene in One Carbon Metabolic (OCM) pathway that affects epigenetic phenomenon through the release of free methyl groups during the irreversible reduction of 5,10-methylenetetrahydrofolate to 5-methyltetrahydrofolate [7]. *MTHFR* C677T gene polymorphism is reported to reduce the enzymatic activity of *MTHFR* gene, resulting in decreased 5-methyl-THF concentrations, increased homocysteine concentrations, and reduced methylation capacity due to non-availability of free methyl groups [8]. The availability of these methyl groups is therefore influenced by genetic and epigenetic variations in the genes involved in One Carbon Metabolic pathway. The availability of the methyl groups may also decide the activity of other blood pressure regulating genes. Literary evidences support that the *T* allele of *MTHFR* C677T gene polymorphism is associated with global DNA hypomethylation and hypertension. Hypertension, in turn, is also reported to be associated with global DNA hypomethylation [9]. Thus, it may be inferred that there is a complex interplay between global DNA methylation, hypertension and *MTHFR* gene polymorphism and they may be interlinked. *MTHFR* gene is therefore the key gene linked to both hypertension and global DNA methylation (5mC%). While some consider the methylation assay as ‘genome-wide’ methylation, others consider it as global DNA methylation [10], 5mC% is still a cost-effective, high throughput and quantitative technique than genome-wide assay [11].

Studies associating *MTHFR* gene polymorphism and hypertension [5, 12, 13], *MTHFR* gene and methylation [14–17], hypertension and methylation [18–20] independently have been reported. However, no study is found to understand hypertension in terms of both genetic (*MTHFR* C677T) and epigenetic (global DNA methylation) mechanisms. Thus, the present study is an attempt to understand the interrelation between global DNA methylation, *MTHFR* C677T gene polymorphism and hypertension in the selected Mendelian population. The study also tries to understand the relation (if any) between methylation and anti-hypertensive drugs.

## Methods

### Study protocol

The present cross-sectional study is a part of Department of Biotechnology (DBT), Government of India funded research project, where 1634 individuals of either sex in the age group 35–65 years and unrelated upto first cousins were randomly recruited from a single Mendelian population from North India. Blood pressure was recorded on 1536 individuals twice with an interval of ten minutes in each reading using standard gold technique and mercury sphygmomanometer. The study participants were seated comfortably in a chair with the forearm placed at the level of chest on a table while measuring blood pressure. Individuals with chronic hypertension were identified according to 7th Report of the JNC guidelines i.e. systolic blood pressure (SBP) ≥ 140 mmHg and Diastolic Blood Pressure (DBP) ≥ 90 mmHg. Individuals with normal blood pressure, prehypertension, hypertension and low blood pressure were found to be 545, 387, 534 and 70, respectively. Individuals presenting with high SBP and high DBP (N-188) and clinically established hypertensive individuals (N-48), resulting into a total of 236 cases were considered for the present study. A total of 307 individuals that were matched for age and sex for each individual were identified as absolute controls (SBP < 120 mm Hg and DBP < 80 mm Hg). Prehypertensive individuals (SBP ≥ 120 mmHg, < 139 mmHg and DBP ≥ 80 mmHg, < 89 mmHg) were excluded in the present study. Data were also collected on the prescribed drugs (generic names) such as amplodipine, telmisartan etc., from the study participants who were on medication.

Fasting blood samples were collected from the study participants after obtaining written consent. DNA samples were extracted from the blood samples obtained from all the participants [21]. DNA quality check was done using nanodrop spectrophotometer with A_260_/A_230_ < 1.5 as a standard.

### *MTHFR* C677T gene polymorphism

*MTHFR* C677T gene polymorphism was screened on all the DNA samples using PCR–RFLP method [22]. Ten percent of the samples were repeated for genotyping by another individual to cross-check the accuracy during the analysis.

### Global DNA methylation (5mC%) assay

Global DNA methylation levels were analyzed using ELISA based colorimetric technique as per manufacturer’s instructions (MethylFlash™ Methylated DNA Quantification Kit-Colorimetric, Epigentek Group Inc., New York, NY, USA, cat. no. P-1034–96). Briefly, DNA was bound to specifically treated strip wells with high DNA affinity. The 5-methylcytosine of DNA was detected using the antibodies (provided by the manufacturer) and quantified by reading absorbance at 450 nm using spectrophotometer (MultiscanGo Spectrophotometer, Thermo Fisher Scientific, Waltham, Massachusetts, USA) [10]. For each sample, methylation assay was performed with 200 ng DNA in duplicates and the intra- and inter-assay coefficients of variation was seen to be < 5%. Global DNA methylation assay was performed by single individual to avoid handling variation. Global DNA methylation assay results could be obtained only for 218 cases and 263 controls due to quality control and other technical reasons such as low absorbance value or large variations in duplicate results.

### Statistical analyses

Data were entered in MS-Excel and checked twice by two individuals separately to ensure integrity, non-duplication and accuracy of the entire data. Data were presented as median and inter-quartile range (IQR), where the distribution of the variable was not seen to be normal. Mann Whittney and Kruskal–Wallis tests were performed to test for differences in median values for two and more than two groups, respectively. p-value less or equal to 0.05 was considered as statistically significant. Hardy–Weinberg equilibrium for *MTHFR* C677T gene polymorphism was also tested. Statistical analysis was performed using SPSS version 16.0 for windows (SPSS Inc., Chicago, Illinois, USA).

## Results

The present study tries to understand the interrelationship between global DNA methylation (5mC%), *MTHFR* C677T gene polymorphism and hypertension. Differences in the levels of 5mC% were assessed between controls and hypertensive cases (on medication and not on medication). Cases (N-218) and controls (N-263) were compared in terms of their demographic (age, sex), lifestyle (smoking, alcohol consumption, diet), biochemical (folate, vitamin B-12 and homocysteine) and genotypic (*MTHFR* C677T) variables.

In the studied case–control groups, all the confounders for hypertension i.e. age, male sex, smoking, alcohol consumption and non-vegetarian diet were found to be significantly higher among the cases (Table 1).

**Table 1:** Distribution of demographic and lifestyle variables among the hypertensive cases and controls

	Controls	Hypertensive cases	p-value
Age (years) Median (IQR)	45 (38–52)	50 (42–57)	**< 0.01**
Sex			
Male	56 (21.3%)	83 (38.1%)	**< 0.01**
Female	207 (78.7%)	135 (61.9%)	
Smoking			
Yes	122 (47.5%)	127 (58.5%)	**0.02**
No	135 (52.5%)	90 (41.5%)	
Alcohol			
Yes	15 (5.7%)	24 (11.1%)	**0.03**
No	247 (94.3%)	193 (88.9%)	
Diet			
Vegetarian	250 (95.4%)	194 (89.4%)	**0.01**
Non-vegetarian	12 (4.6%)	23 (10.6%)	

The genotypic distribution of *MTHFR* C677T gene polymorphism was found to be similar in cases and controls (p-0.79). The T allele frequency was also found to be similar in both the studied groups. The biochemical parameters i.e. folate, vitamin B-12 and homocysteine that are involved in One Carbon Metabolism and influence global DNA methylation status were also found to be similarly distributed in both cases and controls (Table 2).

**Table 2:** Distribution of biochemical variables and MTHFR C677T gene polymorphism among the hypertensive cases and controls

	Controls	Cases	p-value
Folate [median (IQR)]	3.81 (2.7–6.7)	3.54 (2.6–5.5)	0.30
Vitamin B-12 [median (IQR)]	232 (187.2–326.5)	256 (197–384)	0.09
Homocysteine [median (IQR)]	19.30 (13.4–28.8)	19.40 (13.7–29.1)	0.69
*MTHFR* Genotype			
CC	178 (70.1%)	139 (67.1%)	0.79
CT	65 (25.6%)	58 (28.0%)	
TT	11 (4.3%)	10 (4.8%)	
Hardy–Weinberg p-value	0.291	0.483	
T allele frequency	0.17	0.18	0.738

**Fig. 1:**
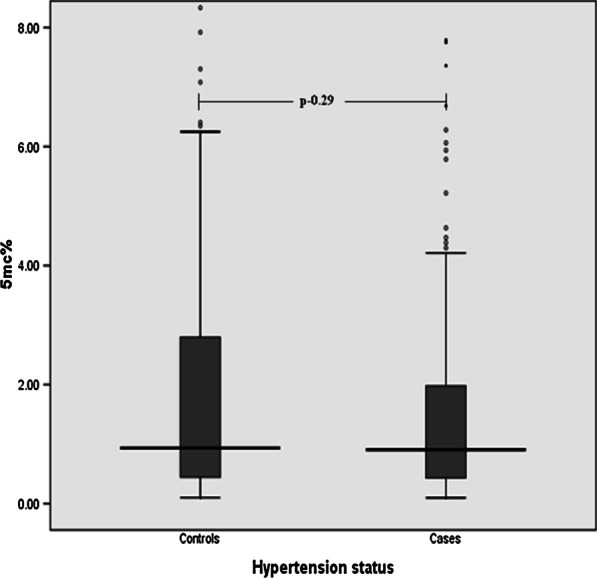
Global DNA methylation in controls and hypertensive cases

Differential global DNA methylation (5mC %) was seen across the selected categories of the studied subjects i.e. controls, cases with hypertension (irrespective of medication) (Fig. 1). Median (IQR) levels of global DNA methylation were seen to be lower in cases compared to the controls. However, these observed differences were not found to be statistically significant when compared to the controls. Similar results were found in regression analysis after adjusting for confounders where no significant association was observed between global DNA methylation and hypertension (r^2^-0.92, p-0.08).

**Fig. 2:**
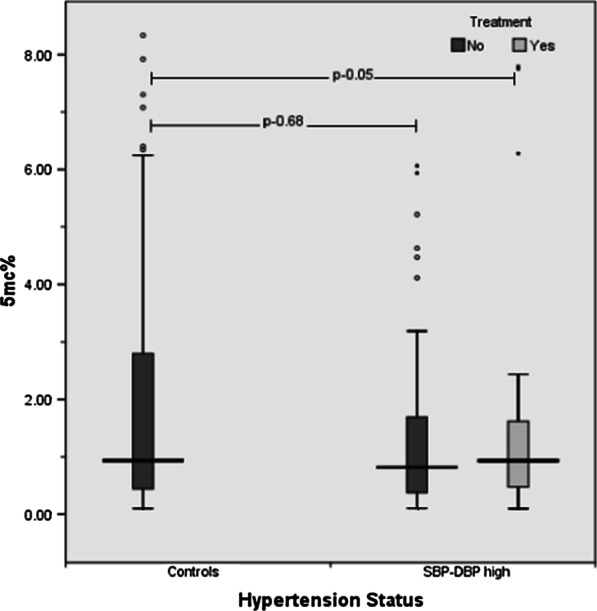
Distribution of 5mC% among controls and hypertensive cases (on treatment and not on treatment) with both high SBP-DBP

The distribution of global DNA methylation levels was then compared between controls and hypertensive cases with respect to treatment (Fig. 2). Global DNA methylation levels was found to be least among cases not on medication [0.82 (0.38–1.70)] followed by cases that were on medication [0.93 (0.47–1.64)] and controls [1.04 (0.49–2.46)]. Only cases that were not on medication had significantly lower global DNA methylation levels compared to the controls (p-0.05). Similar results were found in regression analysis after adjusting for confounders where significant association was observed between global DNA methylation and hypertension with respect to medication (r^2^-0.859, p-0.01).

**Fig. 3:**
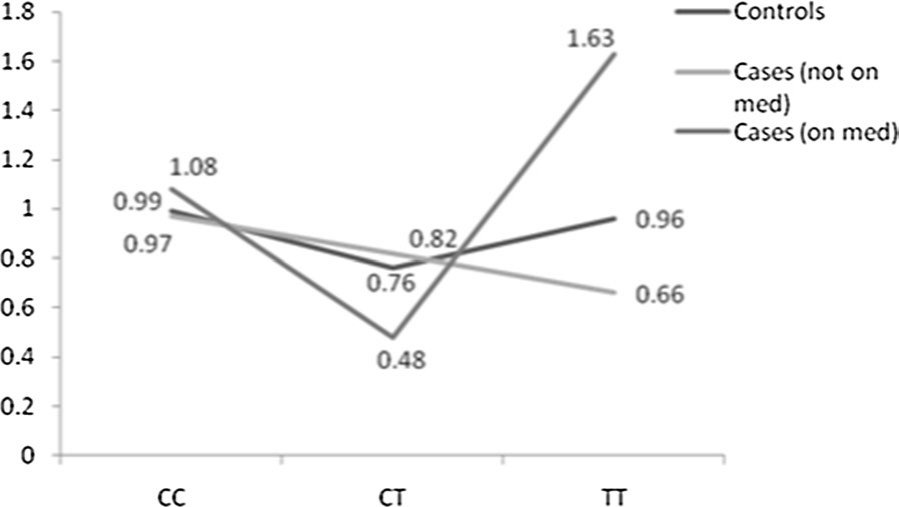
Distribution of 5mC% among hypertensive cases (with/without medication) and controls with respect to *MTHFR* C677T gene polymorphism

Global DNA methylation levels were further compared between controls and cases with respect to *MTHFR* C677T gene polymorphism (Fig. 3). The trend of global DNA methylation seems to vary across the three genotypes i.e. CC, CT and TT in both cases and controls. As expected, among cases (not on medication), a declining trend of global DNA methylation was seen among individuals with CC genotype, followed by CT and TT genotype and this difference was found to be statistically significant (Kruskal–Wallis p-value < 0.001). On the contrary, among controls, a drop in global DNA methylation was seen among CT genotype carrying individuals compared to CC genotype followed by a steep rise among TT genotype carrying individuals. Surprisingly, a similar trend was observed among cases that were on medication, where global DNA methylation levels dropped sharply among CT genotype individuals compared to CC genotype carrying individuals followed by an increase in TT genotype carrying individuals. When the respective genotypes of cases (on medication and not on medication) and controls were compared, median global DNA methylation levels was found to be highest among cases on medication with CC genotype (1.08) followed by controls (0.99) and cases not on medication (0.97), albeit with no statistical significance (p-0.68). Further, with respect to CT genotype, cases on medication exhibited significantly lower median global DNA methylation levels (0.48) compared to cases not on medication (0.76) and controls (0.82) (p < 0.01). However, with respect to TT genotype, median global DNA methylation levels were found to be highest among cases on medication (1.08) followed by controls (0.96) and cases not on medication (0.66) and this difference was found to be statistically significant (p < 0.01).

## Discussion

Hypertension is a multifactorial complex disease that is a result of gene-environment interactions. Interplay between global DNA methylation and *MTHFR* C677T gene polymorphism seems to be important in hypertension. The results of the present study indicate that global DNA hypomethylation is seen in hypertension (irrespective of the treatment) and is not associated with folate, vitamin B-12 and homocysteine. The global DNA methylation levels seem to be significantly dropped in individuals with both SBP-DBP high who are not on medication. Intervention with medication for treatment of hypertension seems to improve methylation levels.

Global DNA hypomethylation has been reported as a consequence of age in numerous studies involving both human and mice [23, 24]. Global DNA methylation has also been reported to be higher among males [25, 26]. In the present study, all the selected demographic (age and sex) and lifestyle (smoking, alcohol consumption and diet) confounders for hypertension were found to be significantly higher among the cases, whereas the biochemical (folate, vitamin B-12 and homocysteine) and genetic (*MTHFR* C677T gene polymorphism) variables were found to be distributed similarly in both the groups. Global DNA hypomethylation was seen among cases even after adjustments for all the confounders for hypertension, albeit with no statistical significance, suggesting that global DNA hypomethylation may be associated with hypertension irrespective of the confounders. Similar findings that associate hypertension with global hypomethylation were reported in previous studies [9, 19, 20, 27].

Global DNA methylation levels were seen to be higher among cases that were on medication compared to cases that were not on medication, or in other words, it may be inferred that global DNA methylation seems to improve after medication among cases. No published literature is available on the influence of antihypertensive drugs on global DNA methylation, but the results of the present study suggest an improvement in methylation levels due to intervention with antihypertensive drugs. The effect of this global DNA hypomethylation on the genes influencing the blood pressure control among hypertensives as also proposed by Smolarek et al., 2010 cannot be overlooked [9]. Though Smolarek et al., 2010 have discussed about the pleiotropic effects of antihypertensive drugs on global DNA methylation, but no correlation was observed between the treatment and global DNA methylation in their study. Being a rural population, the hypertensive individuals in the present study usually visit the local government hospitals where common antihypertensive drugs such as calcium channel blockers like amlodipine or angiotensin receptor blockers (ARBs) like telmisartan are prescribed. These drugs seem to be acting on the blood pressure controlling genes too, as also suggested by Smolarek et al., 2010 [9]. In the present study also it has been observed that anti-hypertensive drugs seem to alter global DNA methylation levels.

Since hypertension with both high SBP-DBP is a severe form of hypertension that predicts cardiovascular risk [28], when individuals with both SBP-DBP hypertension (not on medication) were considered, they seem to be having significantly reduced global DNA methylation compared to their respective controls (Fig. 2). Similar observations were seen after adjusting for the effect of confounders (r^2^-0.859, p-value – 0.01). Global DNA hypomethylation which is an effect of hypertension in turn may lead to other metabolic adversities (such as cardiovascular adversities) [11] due to genomic instability caused by global DNA hypomethylation [29], but may be reduced or reversed through medication. Interestingly, the trend of 5mC% among cases (SBP-DBP high) seemed similar to that of controls whereby global DNA methylation levels were improved among cases on medication. This suggests that medication may improve the global DNA methylation status.

Further, global DNA methylation is also reported to be affected by mutations in the candidate genes specific to ‘one-carbon’ metabolic pathway like that of *MTHFR*. *MTHFR* gene affects the release of free methyl groups in the homocysteine metabolic pathway during the irreversible reduction of 5,10-methylenetetrahydrofolate to 5-methyltetrahydrofolate [7]. *MTHFR* C677T gene polymorphism can reduce the enzymatic activity of *MTHFR*, resulting in decreased 5-methyl-THF concentrations, increased homocysteine concentrations, and reduced methylation capacity [8]. Thus, T allele carrying individuals of *MTHFR* C677T gene polymorphism are likely to exhibit hypomethylation owing to enhanced methyl group acceptance capacity (higher among TT genotype carrying individuals vs CT and CC individuals) [30]. In the present study, when an attempt was made to understand this phenomenon among cases and controls, similar trend of decreased methylation with an increase in T allele dose (TT < CT < CC) among cases (not on medication) was observed. However, such trend was not found among controls and cases (on medication). Global DNA methylation levels were found to be lower among CT genotype carrying individuals compared to CC genotype but an increase in global DNA methylation level was observed with respect to TT genotype in both controls and cases on medication. A study conducted by Arruda et al., 2013 among healthy individuals has also reported global DNA methylation levels in CT genotype carrying individuals to be lower compared to CC and TT genotype carrying individuals [16]. However, the reason for such a trend among CT genotype carrying individuals with respect to global DNA hypomethylation needs to be explored. Further, the variation of global DNA methylation with respect to genotypes seems to be different only among cases (on medication and not on medication) whereas no such difference was observed among controls.

Intervention with medication seems to play an important role in improvement of DNA methylation status. This could probably be due to two mechanisms. First, medication may directly improve methylation levels and the flux of methyl groups may be diverted to the blood pressure controlling genes, thereby activating them to control blood pressure. Second, the drug may directly act on blood pressure control and this may result in the improvement of methylation status.

Further, the intervention with medication is given irrespective of the *MTHFR* genotype status of an individual but there seems to be an improvement in the methylation status only among homozygote individuals on medication with CC and TT genotypes. An abrupt drop in methylation levels among cases on medication with CT genotype is indicative of poor response of these individuals to antihypertensive drugs. Intervention with medication is supposed to normalize the elevated blood pressure levels and therefore the cases on medication seem to be imitating the 5mC% pattern among controls, as also seen in the present study but the exact mechanisms involved in such a phenomenon are yet to be explored. No comprehensive studies are available that confirm the role of *MTHFR* genotype with respect to the effect of drugs and global DNA methylation.

The sample size in the present study is not robust enough to come to major conclusions on the complex interplay between global DNA methylation, *MTHFR* C677T gene polymorphism and hypertension. However, the results of the present study are indicative of an improvement in global DNA methylation levels due to intervention with antihypertensive drugs, which is more pronounced among individuals with CC and TT genotypes. The findings of the present study and previous studies [9, 11] thus seem to build evidence on the association of global DNA hypomethylation with disturbances in cardiovascular system. Further, the mere fact that medication is improving global DNA methylation among cases is a welcoming observation which can be a primer to the interventional strategies adopted in blood pressure regulation.

## Conclusion

In conclusion, the results of the present study indicate a close association between hypertension, *MTHFR* C677T gene polymorphism and global DNA methylation. Moreover, antihypertensive drugs seem to improve methylation among cases which hints towards curative than preventive approach in blood pressure control. Micronutrients such as vitamin B-12, B-9 and B-6 are important precursors for activation of One Carbon Metabolic pathway and improve availability of free methyl groups. Therefore, methylation levels can be improved by enhancing the capacity of this pathway through nutritional interventions (either dietary or supplements) and this may serve as a strategy to prevent hypertension at population level. However, the findings of the present study need to be validated with large epidemiological-longitudinal studies to further strengthen the association between these three variables.

## Strength and limitations

The present study is first of its kind where relation between *MTHFR* gene polymorphism, global DNA methylation and blood pressure was assessed using a field-based population study design. One major strong hold of the study is the consideration of cases with elevated blood pressure (hypertension) who are unaware of their hypertension status and are not on antihypertensive drugs (such as ARBs, calcium channel blockers or β-blockers). The second advantage of such a study design is selection of a Mendelian population with relatively common gene pool from where both cases (hypertensive) and their controls were selected. The data collection was restricted to one district i.e. Palwal of Haryana state, thereby facilitating the geography match between cases and controls. Therefore, by picking up subjects from same ethnic group and geographical region, most of the confounders for hypertension and *MTHFR* C677T gene polymorphism variation like ethnicity (gene pool) and environment (culture, food habits etc.) are being controlled to the maximum in the present study. Also, the major factors that influence methylation levels i.e., folate, vitamin B-12 and homocysteine are also being controlled in the present study.

## Data Availability

The datasets used and/or analyzed during the current study available from the corresponding author on reasonable request.

## Abbreviations

*MTHFR*: Methylenetetrahydrofolate reductase
*5mC%*: Global DNA methylation
*OCM*: One Carbon Metabolic
*SBP*: Systolic blood pressure
*DBP*: Diastolic blood pressure
*IQR*: Interquartile range
